# Supplementary material for: Preparation of Linear Actuators Based on Polyvinyl Alcohol Hydrogels Activated by AC Voltage
Source: Polymers (Basel). 2023 Jun 19;15(12):2739. doi: 10.3390/polym15122739 (PMC10305258; doi:10.3390/polym15122739)
Supplement: Supplementary file 1 [file polymers-15-02739-s001.zip › polymers-2451514-supplementary/supplementary files.docx]

Supplementary

Preparation of linear actuators based on polyvinyl alcohol hydrogels activated by AC-voltage

Tarek Dayyoub^1,2*^, Aleksey Maksimkin^1^, Dmitry I. Larionov^1^, Olga V. Filippova^1^, Dmitry V. Telyshev^1,3^, Alexander Yu. Gerasimenko^1,3^

^1^ Institute for Bionic Technologies and Engineering, I.M. Sechenov First Moscow State Medical University (Sechenov University), Bolshaya Pirogovskaya Street 2-4, 119991 Moscow, Russia; tarekzd@windowslive.com (T.D.); aleksey_maksimkin@mail.ru (A.M.); dmitry.larionov0625@gmail.com (D.I.L.); borisovaolya@yandex.ru (O.V.F.); telyshev@bms.zone (D.V.T.); gerasimenko@bms.zone (A.Y.G.)

^2^ Department of Physical Chemistry, National University of Science and Technology “MISIS”,
119049 Moscow, Russia

^3^ Institute of Biomedical Systems, National Research University of Electronic Technology, Zelenograd, 124498 Moscow, Russia

***** Correspondence: tarekzd@windowslive.com (T.D.)

**Table S1**. Overall contraction and extension, and activation time for P2B2 hydrogels, loaded by ~20 kPa.

| **Type of reinforcement** | **Spiral weave** | | | | | | | |
| --- | --- | --- | --- | --- | --- | --- | --- | --- |
| **Voltage, V** | 90 | | 110 | | 150 | | 200 | |
| **Frequency, Hz** | 50 | 500 | 50 | 500 | 50 | 500 | 50 | 500 |
| **Overall extension, %** | 12.58±0.22 | 13.85±0.52 | 19.66±1.23 | 20.21±1.22 | 31.54±2.58 | 34.58±2.52 | 50.10±1.45 | 52.81±1.33 |
| **Activation time, sec** | 5.85±0.15 | 5.35±0.15 | 5.25±0.20 | 4.95±0.25 | 4.55±0.25 | 4.85±0.15 | 6.31±0.15 | 4.50±0.2 |
| **Type of reinforcement** | **Fabric woven braided mesh** | | | | | | | |
| **Voltage, V** | 90 | | 110 | | 150 | | 200 | |
| **Frequency, Hz** | 50 | 500 | 50 | 500 | 50 | 500 | 50 | 500 |
| **Overall contraction, %** | 2.86±0.10 | 3.21±0.21 | 7.65±1.25 | 10.58±1.54 | 12.55±2.12 | 16.26±1.58 | 20.25±0.85 | 21.24±0.76 |
| **Activation time, sec** | 9.75±1.5 | 5.70±1.0 | 8.50±0.25 | 6.15±0.20 | 6.85±0.25 | 4.25±0.15 | 5.15±0.15 | 4.60±0.10 |

**Table S2**. Overall contraction and extension, and activation time for P2B2 hydrogels, loaded ~40 kPa.

| **Type of reinforcement** | **Spiral weave** | | | | | | | |
| --- | --- | --- | --- | --- | --- | --- | --- | --- |
| **Voltage, V** | 90 | | 110 | | 150 | | 200 | |
| **Frequency, Hz** | 50 | 500 | 50 | 500 | 50 | 500 | 50 | 500 |
| **Overall extension, %** | 9.67±1.25 | 10.55±2.21 | 15.36±2.32 | 17.25±1.89 | 25.36±2.36 | 28.56±1.98 | 41.25±2.39 | 47.59±2.37 |
| **Activation time, sec** | 3.50±0.25 | 2.85±0.25 | 3.15±0.15 | 2.50±0.15 | 2.70±0.20 | 2.35±0.15 | 3.10±0.25 | 2.80±0.15 |
| **Type of reinforcement** | **Fabric woven braided mesh** | | | | | | | |
| **Voltage, V** | 90 | | 110 | | 150 | | 200 | |
| **Frequency, Hz** | 50 | 500 | 50 | 500 | 50 | 500 | 50 | 500 |
| **Overall contraction, %** | 2.15±0.15 | 3.15±0.18 | 6.89±2.32 | 9.48±1.26 | 10.25±1.36 | 13.98±2.28 | 16.93±2.07 | 18.35±3.85 |
| **Activation time, sec** | 12.85±1.50 | 8.65±1.50 | 10.25±1.25 | 7.60±1.25 | 6.55±1.15 | 5.85±85 | 5.80±0.10 | 5.10±0.10 |

**Table S3**. Overall contraction and extension, and activation time for P5B2 hydrogels, loaded by ~20 kPa.

| **Type of reinforcement** | **Spiral weave** | | | | | | | |
| --- | --- | --- | --- | --- | --- | --- | --- | --- |
| **Voltage, V** | 90 | | 110 | | 150 | | 200 | |
| **Frequency, Hz** | 50 | 500 | 50 | 500 | 50 | 500 | 50 | 500 |
| **Overall extension, %** | 15.38±0.22 | 18.66±0.32 | 22.86±0.89 | 23.01±0.76 | 35.77±2.44 | 36.25±2.82 | 50.70±1.58 | 52.68±1.24 |
| **Activation time, sec** | 3.95±0.10 | 3.45±0.15 | 3.15±0.22 | 2.95±0.64 | 3.50±0.20 | 2.80±0.15 | 4.40±0.15 | 2.90±0.10 |
| **Type of reinforcement** | **Fabric woven braided mesh** | | | | | | | |
| **Voltage, V** | 90 | | 110 | | 150 | | 200 | |
| **Frequency, Hz** | 50 | 500 | 50 | 500 | 50 | 500 | 50 | 500 |
| **Overall contraction, %** | 4.30±0.44 | 6.17±0.31 | 8.32±0.15 | 11.85±0.77 | 8.78±0.12 | 13.75±0.23 | 16.75±1.28 | 20.95±1.25 |
| **Activation time, sec** | 4.18±0.30 | 3.38±0.15 | 3.82±0.15 | 3.33±0.15 | 3.90±0.10 | 3.55±0.15 | 4.25±0.15 | 4.0±0.10 |

**Table S4**. Overall contraction and extension, and activation time for P5B2 hydrogels, loaded by ~40 kPa.

| **Type of reinforcement** | **Spiral weave** | | | | | | | |
| --- | --- | --- | --- | --- | --- | --- | --- | --- |
| **Voltage, V** | 90 | | 110 | | 150 | | 200 | |
| **Frequency, Hz** | 50 | 500 | 50 | 500 | 50 | 500 | 50 | 500 |
| **Overall extension, %** | 11.55±0.25 | 15.87±2.56 | 17.98±2.25 | 20.58±1.69 | 28.81±2.58 | 31.55±2.69 | 25.22±1.15 | 27.55±1.25 |
| **Activation time, sec** | 2.35±0.25 | 2.10±0.15 | 1.85±0.05 | 1.45±0.10 | 1.75±0.15 | 1.35±0.10 | 2.80±0.20 | 1.45±0.15 |
| **Type of reinforcement** | **Fabric woven braided mesh** | | | | | | | |
| **Voltage, V** | 90 | | 110 | | 150 | | 200 | |
| **Frequency, Hz** | 50 | 500 | 50 | 500 | 50 | 500 | 50 | 500 |
| **Overall contraction, %** | 3.75±1.23 | 4.89±1.78 | 5.99±1.51 | 9.69±1.36 | 6.15±2.12 | 11.36±1.65 | 14.21±1.15 | 17.11±1.54 |
| **Activation time, sec** | 6.15±0.20 | 5.50±0.25 | 4.85±0.20 | 4.15±0.15 | 4.45±0.20 | 4.10±0.15 | 4.70±0.15 | 4.15±0.15 |

**Table S5.** Overall contraction and extension, and activation time for P7B2 hydrogels, loaded by ~20 kPa.

| **Type of reinforcement** | **Spiral weave** | | | | | | | |
| --- | --- | --- | --- | --- | --- | --- | --- | --- |
| **Voltage, V** | 90 | | 110 | | 150 | | 200 | |
| **Frequency, Hz** | 50 | 500 | 50 | 500 | 50 | 500 | 50 | 500 |
| **Overall extension, %** | 16.28±0.22 | 17.96±0.22 | 20.22±0.22 | 22.58±1.29 | 41.41±0.28 | 42.56±1.28 | 57.5±0.22 | 59.88±1.11 |
| **Activation time, sec** | 3.83±0.08 | 3.38±0.13 | 3.45±0.11 | 3.30±0.05 | 3.40±0.10 | 3.1±0.10 | 3.85±0.05 | 3.25±0.05 |
| **Type of reinforcement** | **Fabric woven braided mesh** | | | | | | | |
| **Voltage, V** | 90 | | 110 | | 150 | | 200 | |
| **Frequency, Hz** | 50 | 500 | 50 | 500 | 50 | 500 | 50 | 500 |
| **Overall contraction, %** | 8.15±0.26 | 9.43±0.27 | 9.42±0.72 | 10.69±0.36 | 11.45±.65 | 13.55±0.78 | 18.10±0.55 | 21.50±0.85 |
| **Activation time, sec** | 4.93±0.07 | 4.53±0.23 | 4.47±0.03 | 4.60±0.25 | 3.75±0.15 | 3.45±0.10 | 3.70±0.05 | 3.20±0.05 |

**Table S6**. Overall contraction and extension, and activation time for P7B2 hydrogels, loaded by ~40 kPa.

| **Type of reinforcement** | **Spiral weave** | | | | | | | |
| --- | --- | --- | --- | --- | --- | --- | --- | --- |
| **Voltage, V** | 90 | | 110 | | 150 | | 200 | |
| **Frequency, Hz** | 50 | 500 | 50 | 500 | 50 | 500 | 50 | 500 |
| **Overall extension, %** | 12.55±1.52 | 13.2±0.25 | 18.88±1.54 | 20.25±2.44 | 35.45±1.55 | 38.32±2.58 | 54.45±2.12 | 55.87±2.54 |
| **Activation time, sec** | 3.1±0.15 | 2.20±0.10 | 2.95±0.10 | 2.65±0.10 | 2.55±0.10 | 2.10±0.20 | 2.2±0.05 | 1.85±0.05 |
| **Type of reinforcement** | **Fabric woven braided mesh** | | | | | | | |
| **Voltage, V** | 90 | | 110 | | 150 | | 200 | |
| **Frequency, Hz** | 50 | 500 | 50 | 500 | 50 | 500 | 50 | 500 |
| **Overall contraction, %** | 6.88±1.35 | 7.69±1.65 | 7.26±1.72 | 9.12±1.31 | 9.55±1.29 | 11.54±1.59 | 15.10±0.14 | 19.89±0.22 |
| **Activation time, sec** | 5.55±0.25 | 5.10±0.15 | 4.95±0.25 | 4.75±0.10 | 4.25±0.20 | 3.95±0.25 | 3.55±0.5 | 3.45±0.05 |

**Table S7**. Overall contraction and extension, and activation time for P10B2 hydrogels, by ~20 kPa.

| **Type of reinforcement** | **Spiral weave** | | | | | | | |
| --- | --- | --- | --- | --- | --- | --- | --- | --- |
| **Voltage, V** | 90 | | 110 | | 150 | | 200 | |
| **Frequency, Hz** | 50 | 500 | 50 | 500 | 50 | 500 | 50 | 500 |
| **Overall extension, %** | 8.22±1.11 | 20.55±1.78 | 24.11±0.56 | 30.11±1.89 | 40.65±3.87 | 51.42±4.55 | 60.02±3.12 | 61.89±4.38 |
| **Activation time, sec** | 4.60±0.35 | 3.97±0.40 | 4.18±0.40 | 3.42±0.40 | 3.85±0.20 | 3.41±0.25 | 4.40±0.20 | 3.75±0.25 |
| **Type of reinforcement** | **Fabric woven braided mesh** | | | | | | | |
| **Voltage, V** | 90 | | 110 | | 150 | | 200 | |
| **Frequency, Hz** | 50 | 500 | 50 | 500 | 50 | 500 | 50 | 500 |
| **Overall contraction, %** | 6.42±0.84 | 10.55±2.07 | 10.04±2.40 | 13.50±0.35 | 12.45±1.25 | 15.21±0.23 | 19.33±1.25 | 20.58±1.11 |
| **Activation time, sec** | 2.85±0.20 | 2.63±0.28 | 2.55.70±0.65 | 2.25±0.20 | 2.45±0.20 | 2.15±0.15 | 3.10±0.10 | 2.50±0.05 |

**Table S8.** Overall contraction and extension, and activation time for P10B2 hydrogels, by ~40 kPa.

| **Type of reinforcement** | **Spiral weave** | | | | | | | |
| --- | --- | --- | --- | --- | --- | --- | --- | --- |
| **Voltage, V** | 90 | | 110 | | 150 | | 200 | |
| **Frequency, Hz** | 50 | 500 | 50 | 500 | 50 | 500 | 50 | 500 |
| **Overall extension, %** | 7.45±0.23 | 18.54±2.22 | 21.45±0.36 | 25.59±3.22 | 35.56±2.65 | 44.85±3.47 | 51.14±2.57 | 55.50±2.19 |
| **Activation time, sec** | 2.30±0.25 | 2.15±0.20 | 2.55±0.25 | 2.15±0.10 | 2.35±0.15 | 2.05±0.10 | 2.85±0.15 | 2.35±0.10 |
| **Type of reinforcement** | **Fabric woven braided mesh** | | | | | | | |
| **Voltage, V** | 90 | | 110 | | 150 | | 200 | |
| **Frequency, Hz** | 50 | 500 | 50 | 500 | 50 | 500 | 50 | 500 |
| **Overall contraction, %** | 4.88±0.56 | 7.54±1.28 | 5.65±1.21 | 8.98±1.36 | 9.55±1.34 | 13.45±1.32 | 18.77±1.57 | 19.78±1.22 |
| **Activation time, sec** | 3.25±0.20 | 3.05±0.15 | 3.10±0.15 | 2.75±0.20 | 2.85±0.20 | 2.55±0.20 | 3.70±0.10 | 2.80±0.15 |


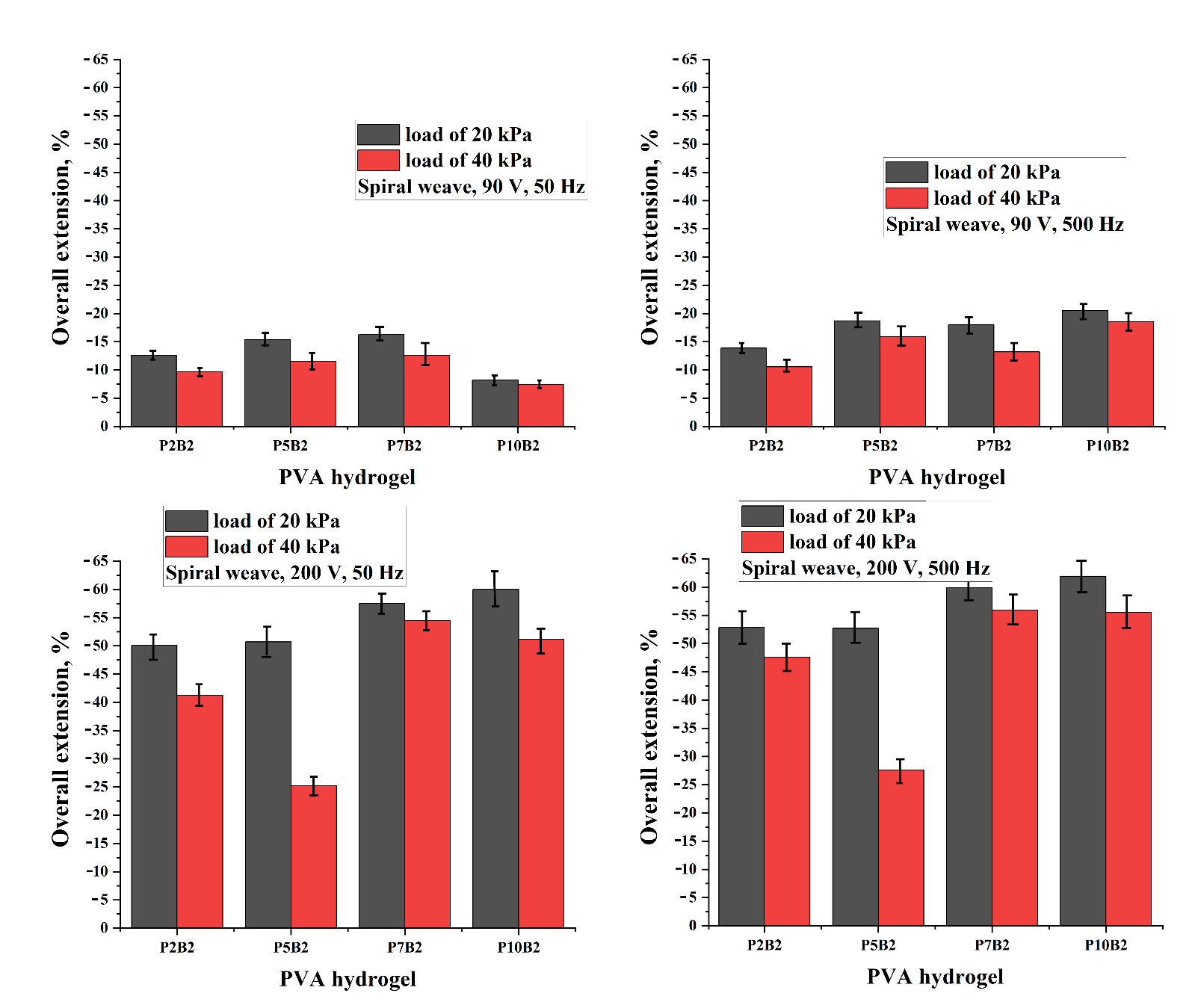


**Figure S1**. Overall extension of PVA hydrogels reinforced by spiral weave under different values of AC-voltage, frequency, and load.


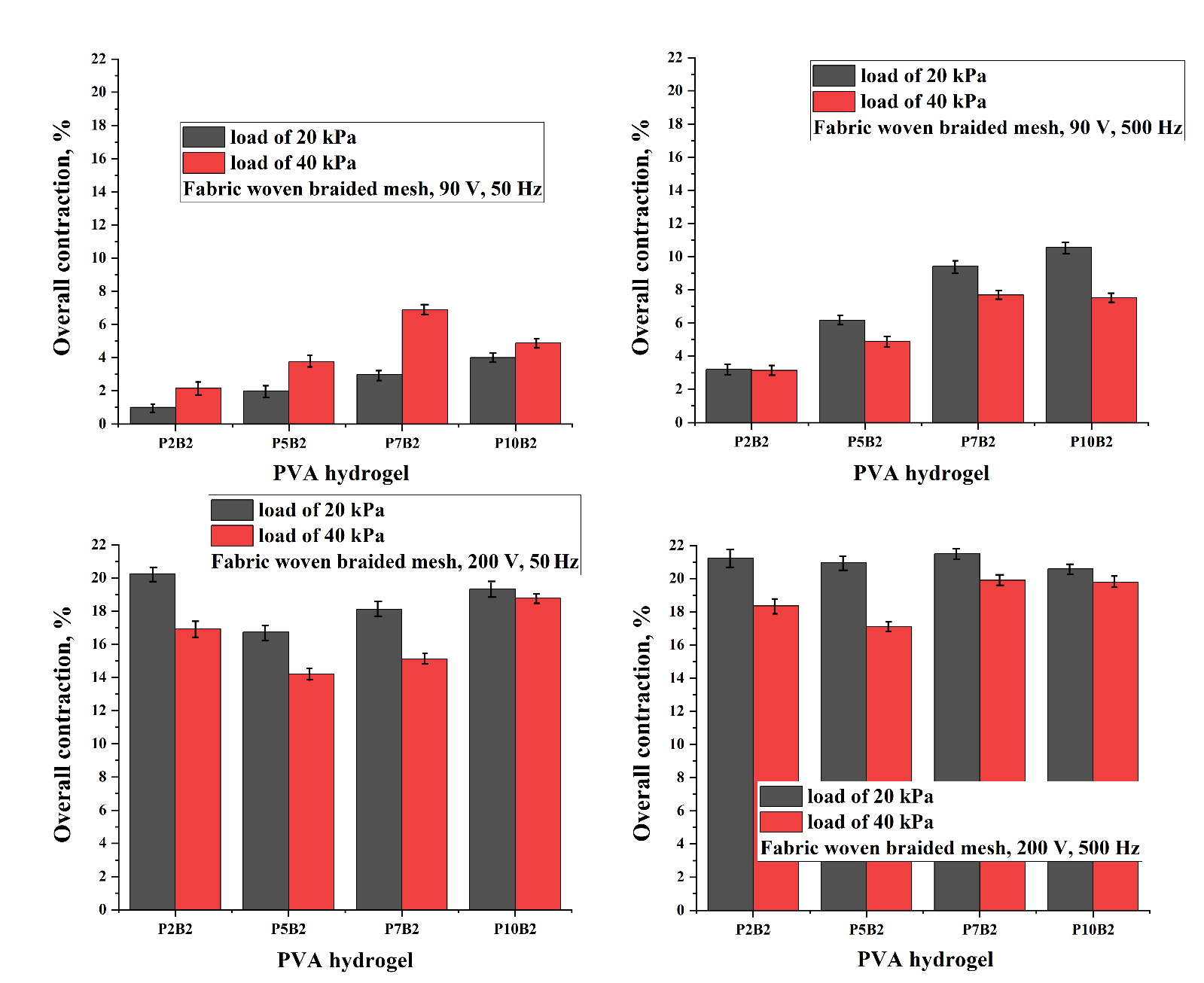


**Figure S2**. Overall contraction of PVA hydrogels reinforced by fabric woven braided mesh under different values of AC-voltage, frequency and load


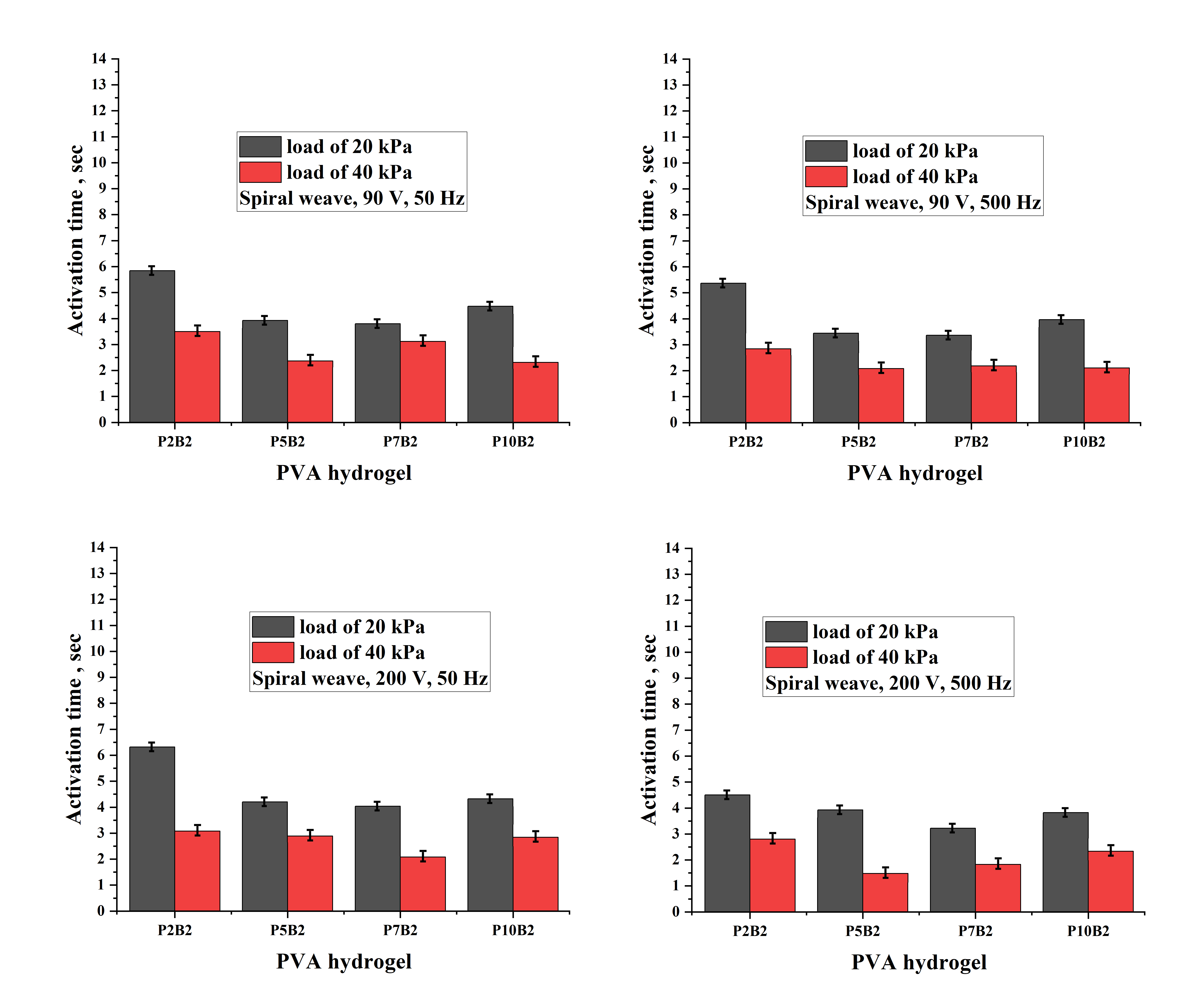


**Figure S3**. Activation time of PVA hydrogels reinforced by spiral weave under different values of AC-voltage, frequency and load


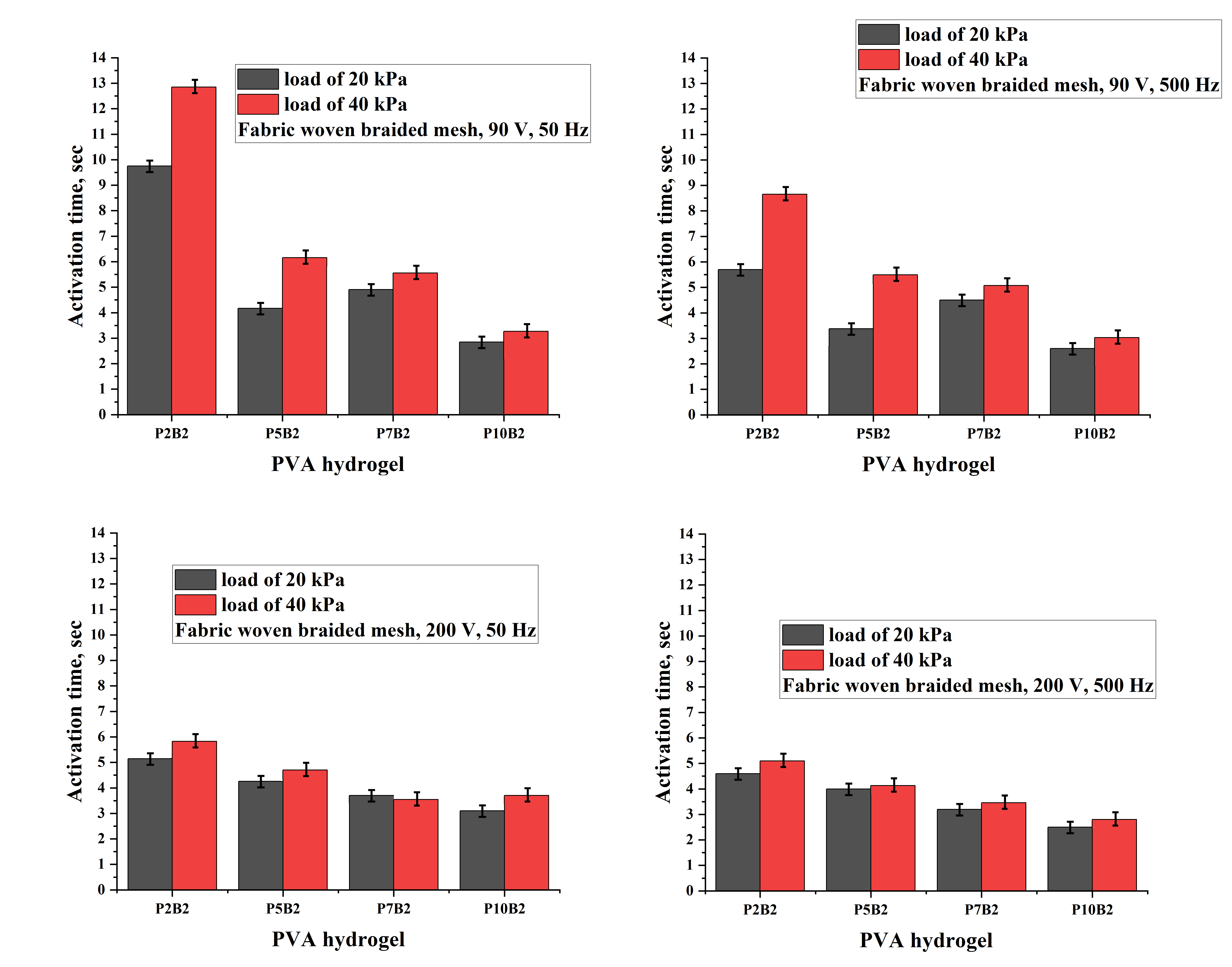


**Figure S4**. Activation time of PVA hydrogels reinforced by fabric woven braided mesh under different values of AC-voltage, frequency, and load.
